# How methodological cues shape trust in psychological research and legal judgements: a vignette study

**DOI:** 10.1098/rsos.260648

**Published:** 2026-10-21

**Authors:** Sera Wiechert, Elise de Groot, Bastiaan T. Rutjens, Yoni Pertzov, Bruno Verschuere

**Affiliations:** ^1^ Department of Criminology, Max Planck Institute for the Study of Crime Security and Law, Freiburg, Germany; ^2^ Department of Clinical Psychology, University of Amsterdam, Amsterdam, The Netherlands; ^3^ Department of Social Psychology, University of Amsterdam, Amsterdam, The Netherlands; ^4^ Department of Psychology, Hebrew University of Jerusalem, Jerusalem, Israel

**Keywords:** open science, memory, preregistration, meta-analysis, replication

## Abstract

Over the past decade, psychology has embraced transparency and reproducibility reforms to address the replication crisis, raising the question of whether these signals help legal decision makers identify reliable psychological evidence. This study examines how these reforms influence perceived study reliability and downstream decision-making. We hypothesized that higher methodological quality (larger sample sizes, preregistration, and replications or meta-analyses) would increase perceived reliability and influence guilt judgements. We conducted an online vignette experiment, including individuals with doctorates and laypersons, who evaluated fictitious legal cases accompanied by research summaries varying in sample size (low versus high), preregistration status (preregistered versus not) and study design (primary study versus replication versus meta-analysis). Larger sample sizes consistently enhanced perceived reliability across both groups, whereas preregistration did not. Doctorate participants rated replications and meta-analyses as more reliable than primary studies; laypersons did not differentiate among designs. Importantly, none of the methodological quality cues influenced guilt judgements, despite shaping reliability judgements. This suggests that recognizing methodological quality alone may not shape downstream decisions. Future research should replicate the evidence in more ecologically valid legal designs (e.g. expert testimony) and with a broader range of methodological indicators to better understand how methodological quality influences perceived reliability and applied reasoning.

## 1. Introduction

Over the past decade, psychology has undergone a shift towards greater transparency and reproducibility. Sparked by the replication crisis in the early 2010s—which revealed that a substantial amount of influential findings could not be replicated (e.g. [1,2])—the field began to critically reassess its methodological foundations. This period of reflection prompted a collective effort to restore trust in psychological science and strengthen the reliability of its evidence base. Two complementary reform streams emerged in response: one aimed at reassessing existing knowledge, and the other at improving research practices for contemporary research.

The first stream focused on evaluating the robustness of existing psychological evidence through systematic replication efforts and meta-analyses, although their proportions remain low [3]. This is critical, as individual primary studies are generally considered weaker sources of evidence than replications and meta-analyses, which evaluate robustness and cumulative evidential strength across multiple studies. Notably, the quality of the cumulative evidence heavily relies on the quality of the underlying literature: meta-analyses that aggregate studies affected by questionable research practices or publication bias risk amplifying rather than correcting evidence distortions (e.g. see [4]). While such approaches predate the replication crisis, they have gained renewed prominence as tools for quantifying effect reliability and generalizability. Large-scale collaborative replication projects have provided valuable insights into the replicability of central psychological findings (e.g. [1,5]) as well as legal and forensic psychology more specifically (e.g. [6,7]), while recent meta-analyses have documented discrepancies between original and replicated effect sizes (e.g. [8,9]). Together, these initiatives aim to provide a more accurate estimate of evidential strength in the psychological literature.

The second reform stream has sought to improve contemporary research through enhanced methodological transparency and rigour, collectively referred to as Open Science practices [10]. These aim to make all stages of the research process open, accessible and verifiable. They include open access publishing, sharing research materials, data and code, and preregistering study protocols [11]. Whereas open access and resource sharing primarily increase transparency and accessibility, preregistration is intended to strengthen rigour by constraining researchers’ degrees of freedom and mitigating biases such as p-hacking (i.e. manipulating analyses or data until statistically significant results are obtained), HARKing (i.e. hypothesizing after results are known) and selective reporting (i.e. reporting only favourable or significant results while omitting others; [12]). It is worth noting, however, that preregistration does not add rigour by itself; rather, it allows others to transparently evaluate how severely a claim has been tested and how likely a test was to reveal a hypothesis as false had it been false [13]. A vague or uninformative preregistration, or one from which researchers substantially deviate without justification, therefore offers little more protection against bias than no preregistration at all. Consistent with this, recent empirical comparisons of preregistered and non-preregistered studies in psychology have found that preregistration does not necessarily predict greater replicability of findings [14], indicating a gap between what preregistration is designed to achieve and its demonstrable effects in practice.

### 1.1. Evaluating the progress

More than a decade after these reforms were introduced, their adoption has been evaluated across several research fields, though not always within psychology specifically. Chin *et al.* [15], for instance, examined Open Science indicators in the empirical legal studies literature. Within this sample, publication accessibility was widespread (over 80% of articles openly available), whereas data sharing and preregistration, despite steady growth, remain less common [15]. Hardwicke *et al.* [16–18] similarly tracked these practices across the social sciences more broadly, while Louderback *et al.* [19] focused specifically on the gambling studies literature. Overall, they found that while Open Science practices are gaining traction, uptake varies across subfields and practices.

Specifically, within psychological fields, similar patterns emerge. A recent review of Open Science practices in the false memory literature [3] revealed a similar pattern of gradual but uneven progress. Across 388 studies published between 2010 and 2023, nearly 87% implemented at least one Open Science practice, with open publication access being the most consistently adopted. Data sharing showed the strongest increase, reaching approximately 75% by 2023, whereas preregistration and analysis script sharing lagged behind at around 20–25%. Replications and meta-analyses also remained rare. Overall, the literature depicts a field in transition: one where accessibility is increasingly common, while more practices that enable reproducibility and evidence evaluation (e.g. preregistration, replication and meta-analysis) are still emerging.

### 1.2. From adoption to recognition

As Open Science reforms become more widely adopted, the question arises whether they are also recognized and valued as indicators of scientific quality. In other words, beyond improving research practices procedurally, do these practices affect how scientific findings are perceived? If transparency and rigour are meant to strengthen reliability, it is essential to determine whether these features also function as recognizable signals of reliability to researchers and the public alike.

People's confidence in scientific work has often been shaped by social cues such as perceived expertise, moral alignment or institutional reputation [20]. Individuals can also be sensitive to methodological indicators of quality. Classical research norms, such as sample size, for instance, have served as cues of evidential strength, with larger samples seen as producing more reliable findings among science journalists [21]. Sensitivity to methodological quality has also been studied in legal contexts specifically, where research suggests that judges and jurors varied in their ability to evaluate the quality of evidence and detect threats to its validity [22,23].

By contrast, Open Science indicators are relatively new and potentially less intuitively connected to study quality for both specialists and non-specialists. Thus, the effectiveness of these reforms partly depends on whether transparency cues are understood and valued as indicators of rigour. Assessing whether Open Science practices are acknowledged as reliable signals—within and beyond the scientific community—is therefore crucial for evaluating the societal reach of methodological reform.

Building on this idea, recent research has begun to examine whether contemporary transparency practices function as signals of reliability across diverse psychological fields within differing populations. Some experimental studies found that transparency cues increased public trust in science, while others reported null or even negative effects. For instance, Field *et al.* [24] manipulated preregistration information in study descriptions but, owing to a failed manipulation check, drew no firm conclusions. In general population samples, Wingen *et al.* [25] showed that informing participants about methodological reforms did not mitigate reduced trust following replication failures, and Anvari & Lakens [26] similarly found that information about questionable research practices decreased trust. In contrast, Rosman *et al.* [27] reported that openly sharing study materials, data and code enhanced trust in science across two large general population German samples, although effects were less consistent in follow-up work. Likewise, Schneider *et al.* [28] demonstrated that open-science badges signalling transparency increased trust among students and academics but had weaker effects among the general public. Finally, Song *et al.* [29] showed across three preregistered experiments that both academic and general audiences rated Open Science–adhering researchers and studies as more reliable than those that did not.

Taken together, prior findings suggest that transparency cues can enhance perceptions of scientific reliability when they are visible and interpretable. However, existing evidence remains fragmented, often focusing on general trust in science rather than on how specific methodological features are evaluated when scientific evidence is used to inform concrete, real-world-relevant decisions. Moreover, the populations studied vary widely—from scientists and students to journalists and the general public—limiting direct comparisons across audiences and contexts.

### 1.3. The present research

The present research addresses this gap by examining whether contemporary indicators of methodological quality influence how psychological evidence is evaluated when presented in a legal setting. Specifically, we tested whether research norms that differ in epistemic function—preregistration (enhancing methodological rigour) and study design (primary study, replication or meta-analysis; reflecting evidential hierarchy)—affect not only perceived reliability of research but also whether that evidence is used in downstream decision-making about guilt. Notably, replication studies and meta-analyses are generally considered stronger sources of evidence than single studies, as they test the robustness and generalizability of pre-existing evidence (e.g. [30,31]). Preregistration enhances transparency and reliability by constraining researcher degrees of freedom (e.g. p-hacking and HARKing) and reducing the potential of publication bias [12].

To contextualize these contemporary indicators, we additionally manipulated sample size, a classical cue of evidential strength [21]. This inclusion served two purposes: conceptually, to benchmark newer methodological norms against a well-established quality signal; and methodologically, as a manipulation check, to verify that participants—especially those with research experience—recognized traditional indicators of study quality before interpreting effects of less familiar ones.

#### 1.3.1. Application to legal psychology

We examined these questions in a legally relevant context, focusing on false memory research. Perceived research reliability is especially consequential in this domain, where psychological evidence directly informs legal judgements, courtroom testimony and investigative procedures (e.g. [32–35]). Scientific findings in legal contexts should be both transparent and interpretable to non-experts, as their consequences extend to anyone interacting with the justice system—as jurors, witnesses or suspects. False memory research therefore offers a unique opportunity to examine whether methodological quality shapes both scientific reliability and further consequences such as legal judgements. In addition, as the recent review [3] showed, false memory research exemplifies both the public relevance and the uneven methodological maturity of psychological subfields. This combination makes it a useful test case for examining whether methodological quality cues influence perceptions of scientific reliability and downstream legal evaluations.

#### 1.3.2. Overview of the present study

We examined whether contemporary methodological features of psychological studies might affect both the perceived reliability of scientific findings and corresponding legal judgements. Participants evaluated legal case information accompanied by short, abstract-like research summaries whose methodological features were systematically manipulated: study design (primary versus replication versus meta-analysis), preregistration (yes versus no information) and sample size (low versus high), allowing us to assess whether methodological quality cues affected confidence in scientific findings and judgements of guilt. Notably, the reported research summaries always presented findings pointing to the potential of suspect innocence. The study is therefore best characterized as a controlled test of sensitivity to methodological quality cues, with the legal case serving as an applied judgement context rather than a simulation of trial procedure. We acknowledge that the vignette paradigm necessarily abstracts away from the conditions under which jurors encounter and evaluate evidence at trial (i.e. in terms of complexity and procedural elements). For example, participants’ decisions carry no real consequences, occur without deliberation or adversarial argumentation and involve simplified written summaries. The findings should therefore be understood as a first step towards understanding how methodological quality cues shape evaluations of psychological evidence, rather than as direct evidence about juror behaviour in legal proceedings. Accordingly, the guilt measure is informative about whether cue sensitivity extends to a downstream applied judgement within this task, but not about whether methodological quality influences verdicts under trial conditions.

We preregistered six hypotheses. First, we tested the effect of sample size. To this end, we hypothesized that participants would report more confidence in the research finding (manipulation check; Hypothesis 1) and would be less likely to find the suspect guilty when studies use large sample sizes (versus small; Hypothesis 2). Second, we tested the effect of preregistration status. We hypothesized that participants would report more confidence in the research finding (Hypothesis 3) and would be less likely to find the suspect guilty when studies were preregistered (versus not preregistered; Hypothesis 4). Finally, we tested the effect of study design. We hypothesized that participants would report more confidence in the research finding (Hypothesis 5) and would be less likely to find the suspect guilty when studies were replications or meta-analyses (versus primary studies; Hypothesis 6). Hypotheses were restricted to main effects because the existing literature offered insufficient basis for directional predictions about interactions between methodological cues. Interactions were nonetheless estimable within the factorial design and treated as exploratory.

## 2. Method

### 2.1. Transparency and openness

We adhered to the Journal Article Reporting Standards (JARS) guidelines. All data, analysis code and research materials are available at OSF [36]. Data were analysed using *RStudio* and *JASP*. The studies were preregistered (including the analysis plan) at https://osf.io/68gte/overview and https://osf.io/ntkw5/overview. As a deviation from the preregistration, we use the term ‘reliability’ rather than ‘credibility’ throughout this manuscript. No other deviations from the preregistration are reported. Ethics approval was obtained by the Ethics Review Board of the University of Amsterdam (FMG-6066_2024). Participants were presented with a digital consent form detailing the study's purpose, procedures, risks and benefits. They indicated their consent by clicking ‘next’ to proceed to the study. *ChatGPT* v. 5.2 [37] was used at parts of the article to increase the flow and conciseness of the written article. At no point did *ChatGPT* generate new writing and was solely used for editing purposes. This applies to all reported work in this article. In the following, we report how we determined our sample size, all data exclusions, all manipulations and all measures used in the study.

### 2.2. Design

The research comprised two experimental designs (Studies 1 and 2). Study 1 manipulated preregistration status (preregistered versus no information) and sample size (low, approx. 10 participants versus high, approx. 1000 participants), resulting in a 2 × 2 design with four within-subject conditions. Study 2 employed a similar design but manipulated study design (primary study versus replication versus meta-analysis) and sample size (low versus high), resulting in a 3 × 2 design with six within-subject conditions. In both studies, participants rated (i) their confidence in the presented study's findings and (ii) the suspect's guilt, each assessed with a single continuous-response item on a 100-point scale.

Importantly, the decision to distribute the manipulations across two studies was made to limit participant burden. A single design crossing all three factors would have required 12 vignettes per participant, raising concerns about habituation and declining engagement over the course of the task. Distributing the factors across two studies kept the number of vignettes to a manageable four or six per participant. Sample size was included in both studies given its role as a manipulation check for sensitivity to methodological quality cues.

Further, to capture differences in research literacy, we recruited two populations: individuals with completed doctorates (expected to be more familiar with scientific research methodology and therefore more sensitive to methodological quality cues) and lay participants from the general public (excluding doctorates). Hence, Studies 1 and 2 were conducted within two populations separately, yielding four studies but two study designs. The two populations were preregistered and analysed separately to reflect the sequential nature of the study design. The doctorate studies were conducted first, with the primary aim of establishing whether participants would detect methodological quality cues at all, using the sample size effect as the manipulation check. After confirming this check, we proceeded to extend the study to lay participants, applying an identical design.

### 2.3. Sample

Participants completed a 15-minute online session hosted on *Qualtrics* and recruited via *Prolific* (compensation ≈ €2). Inclusion criteria required being at least 18 years old and fluent in English. Importantly, for the layperson studies, half of the participants were recruited from WEIRD (Western, educated, industrialized, rich, democratic) countries with (partial) jury systems (the United States, the United Kingdom, Canada, Germany and France) and half from non-WEIRD countries with comparable systems (Mexico, Brazil, Japan, South Korea and Russia). Recruitment criteria were implemented through *Prolific*’s prescreening filters.

Although our primary analyses were Bayesian, the sample size justification was based on a frequentist power analysis. Specifically, in a previous study, Bottesini *et al.* [21] observed an effect of sample size on confidence ratings among science journalists (*B* = 0.73 on a –3 to +3 scale, *t* = 12.44, *N* = 181). Converting this to a standardized effect size using *d* = *t*/√*N* yields *d* = 0.93 (*f* = 0.46), confirming the large effect. Given that individuals with doctoral training are likewise expected to recognize the importance of sample size in evaluating research, we used this finding as a benchmark for estimating the minimum required sample. Because replication studies typically show a 50% reduction in effect size (i.e. *f*/2 = 0.23 [1]), we decided to remain conservative and assumed a medium effect instead as the basis of our minimum sample calculation (*f* = 0.15). With an *α* = 0.05, power = 0.90, *r* among repeated measures = 0.50 and nonsphericity correction *ε* = 1.00 in G*Power for repeated-measures ANOVA (i.e. within factors [38]),^1^ this indicated that 81 participants would be sufficient for Study 1 and 63 participants for Study 2. For preregistration and study design effects, where effect sizes were unknown, we equally expected small-to-moderate effects (*f* = 0.15). To ensure identical operationalization across populations, the layperson studies employed the same sample size as the doctorate studies.

Each study included 100 participants, resulting in a total of 400 participants across four studies. Participants were excluded if they failed to complete the experiment or failed at least two of four attention checks. Table 1 summarizes the sample characteristics of all studies. Across all studies, sample sizes ranged from 89 to 91 participants after exclusions. Gender distribution was broadly comparable across samples, though Bayesian tests of equal proportions (H0: male = female) were inconclusive in three samples (Study 1 doctorate: BF_01_ = 2.13; Study 1 lay: BF_01_ = 3.82; Study 2 doctorate: BF_01_ = 2.78), and Study 2 lay showed a notable male skew (53 males versus 35 females) with inconclusive evidence in either direction (BF_10_ = 1.44). The mean age was lower in the lay samples than in the doctorate samples (BF_10_ = 101.16), reflecting differences in educational attainment. Among doctorate samples, the proportion residing in jury system countries did not differ significantly (BF_01_ = 3.91); lay samples were recruited exclusively from jury system countries by design. Prior jury experience was relatively uncommon (fewer than 12% in each), with strong evidence of no difference in participation rates between groups (BF_01_ = 665.94). Among lay participants, WEIRD and non-WEIRD country representation was balanced between Studies 1 and 2 (BF_01_ = 5.36). These demographic characteristics are reported descriptively to characterize sample composition, rather than as evidence bearing on the confirmatory analyses, which were conducted separately within each sample. These characteristics may nonetheless have effects on outcomes, and future research directly comparing outcomes across samples should take them into account. Note that the four samples may differ on additional, unobserved or unobservable dimensions beyond those reported, limiting causal inferences in the observed effects.

**Table 1.** Sample size and sample characteristics of all studies. Raw numbers are reported in the table. Specifically, we report the total number of individuals who participated (total participation), the final sample remaining after exclusion criteria were applied (final sample), the distribution of males, females and other genders (gender), the mean and standard deviation of age (age), the number of participants residing in a country with jury court systems (jury system country, e.g. the United States), the number of participants having participated in a jury (prior jury participation) and the number of participants residing in WEIRD and non-WEIRD countries (country category).

	Study 1		Study 2	
	doctorate	lay	doctorate	lay
total participation	99	100	101	100
final sample	91	89	89	91
gender (males, females, other)	51, 40, 0	43, 45, 1	48, 40, 1	53, 35, 3
age (*M,* s.d.)	38.80 (10.75)	35.12 (10.19)	40.65 (12.77)	34.23 (9.87)
jury system country	72	89	76	91
prior jury participation	6	7	10	8
country category (WEIRD, non-WEIRD)	not measured	43, 46	not measured	45, 46

### 2.4. Materials

#### 2.4.1. Vignettes

Participants were presented with a series of fictional legal case vignettes, each followed by an abstract-like summary of a memory research study relevant to the case. Please see the following as an example: *Jordan is on trial in court for drug trading. The police questioned Liam, Jordan's 16-year-old son, who was seen with Jordan during encounters with suspected drug dealers. During the interview, initially silent, Liam later confirmed Jordan's involvement. Jordan's lawyer challenges Jordan's guilt and argues that Liam's testimony may be unreliable owing to suggestive questioning during his interview. He bases this argument on recent research showing how suggestive questioning of minors can bias witness memory and testimony accuracy. Below is an overview of the journal article the lawyer refers to: ‘Researchers conducted a replication study on the impact of suggestive questioning on memory accuracy and witness credibility. In the study, a sample of 1006 participants watched a mock theft movie. Subsequently, half of the participants were interviewed with suggestive questions, while the other half were interviewed with neutral questions. This replication study again found that participants were less accurate and had more false memories after facing suggestive questioning. This finding underscores the influence of suggestive questions on testimony credibility.’*

Importantly, the reported study within the vignette always presented findings pointing to the potential innocence of the suspect (i.e. exonerating evidence). Additionally, for all presented research within the vignette, the reported findings always aligned with the study's hypotheses. The scenarios were designed to simulate nuanced legal situations involving memory-related evidence, thereby capturing the complexity of memory research and enabling a controlled assessment of how research quality potentially influences judgements of trustworthiness and legal outcomes.

The vignettes were systematically manipulated to vary in sample size, study design and preregistration status, allowing for precise control of experimental conditions while maintaining internal consistency. Notably, we did not include direct per-vignette manipulation checks on the methodological manipulation (e.g. ‘Was this study preregistered?’). This was done to avoid sensitizing participants to the manipulated features, which could otherwise inflate effects through demand characteristics. Instead, the effect of sample size (i.e. a well-established cue) served as a sensitivity check across studies, indicating that participants were sensitive to methodological information in the vignettes.

To minimize stimulus-specific biases, we created six distinct case topics, each adapted across all combinations of the independent variables. All six topics concerned false memory research but varied in their focus within the domain (e.g. the misinformation effect and suppressed memories), ensuring thematic consistency while reducing reliance on any single stimulus. In Study 1, this resulted in 16 unique vignettes organized into four sets, with each set featuring one vignette topic presented under the four conditions of the 2 × 2 design. In Study 2, 36 unique vignettes were generated and organized into six sets, each representing one vignette topic presented under the six conditions of the 3 × 2 design. Participants were randomly assigned to one set in each study, and the vignettes were presented in randomized order across participants. All materials are available at OSF [36].

#### 2.4.2. Demographic and exploratory measures

Participants provided demographic information, including age, ethnicity, gender, employment field, job title, background in science or legal studies, and frequency of encountering scientific research in daily life. Additionally, several exploratory questions assessed participants’ prior knowledge and experience related to legal proceedings and research practices, including country of residence with a jury system, prior jury service, perceived importance of replication, meta-analysis and preregistration and factors considered when evaluating lawyer arguments. A complete list is available at OSF [36].

### 2.5. Procedure

The study was conducted online in English. Participants were recruited through *Prolific* and directed to the *Qualtrics* survey platform. After reading the information letter and providing digital consent, participants were presented with a glossary that explained the manipulated concepts in accessible terms. This glossary familiarized participants with our manipulated methodological concepts alongside several distractor terms, ensuring a basic understanding of the terminology used throughout the study. Importantly, the decision to provide methodological explanations in a standalone glossary was deliberate. In a within-subject design where participants encounter multiple cases, re-explaining the same concept across vignettes would have been repetitive, potentially signalling the study's focus to participants. To ensure that participants had genuinely processed the glossary information before proceeding, extensive comprehension checks followed; participants could not advance without answering these correctly. Overall, this approach mirrors how jurors are typically instructed prior to trial (i.e. receiving conceptual background before evaluating cases) and reduces the risk that within-stimulus explanations would prime participants towards the manipulated features.

Following the glossary, participants were informed that they would evaluate a series of fictitious criminal cases and were asked to imagine themselves serving as jury members. They were then presented with four vignettes in Study 1 and six in Study 2. For each vignette, participants rated their confidence in the presented research findings and their judgement of the suspect's guilt. Two attention checks were included after the first and last vignette of each study – one assessing attention to the legal case and the other to the research information. After completing all vignettes, participants answered the demographic and exploratory questions, were debriefed, thanked for their participation and compensated.

### 2.6. Statistical analyses

Confirmatory analyses were conducted on the final total sample after listwise exclusions. Participants who did not complete the survey or who failed at least two of four attention checks were excluded. Exclusion rates ranged from 8.10% (Study 1, doctorate) to 11.90% (Study 2, doctorate) and from 9.00% (Study 2, lay) to 11.00% (Study 1, lay; see table 1). Because participants with incomplete responses were removed prior to analysis, no missing values remained within the final analytic dataset. For all analyses, corresponding effect sizes were reported. The analyses were performed separately for the doctorate and layperson samples.

As preregistered, all analyses followed a Bayesian hypothesis testing approach. Within both study populations, two Bayesian repeated-measures ANOVAs were conducted using JASP's uniform default prior, separately for each dependent variable: (i) confidence in the research findings and (ii) guilt ratings. In Study 1, preregistration status (preregistered versus no information) and sample size (low versus high) were included as within-subject factors. In Study 2, study design (primary study versus replication versus meta-analysis) and sample size (low versus high) served as within-subject factors. For each dependent variable, we report three quantities: the Bayes factor for the best-fitting model relative to the null model, the Bayes factor for each effect and, for the two-level factors (preregistration and sample size), a one-sided paired *t*-test examining directional hypotheses, with averaged scores across the relevant conditions computed prior to analysis.

## 3. Results

### 3.1. Confirmatory analyses

Across all four studies, sample size increased perceived reliability but did not affect guilt judgements. Preregistration affected neither outcome in either population, and study design affected reliability among doctorate participants only. Table 2 summarizes the main Bayesian results; detailed findings are reported by study and population below.

**Table 2.** Summary of main Bayesian results across all four studies. For Study 2, main IV contrasts are shown as pri = primary study, rep = replication, MA = meta-analysis, – = not applicable (Study 2 design is three-level).

	Study 1 doctorate	Study 1 lay	Study 2 doctorate	Study 2 lay
effect	preregistration	preregistration	study design	study design
reliability ratings				
ANOVA				
sample size	BF₁₀ = 3.76 × 10²⁰	BF₁₀ = 3.10 × 10⁹	BF₁₀ = 5.33 × 10³⁰	BF₁₀ = 504 800
main IV	BF₀₁ = 2.65	BF₀₁ = 11.90	BF₁₀(Pri-Rep) = 6.82 BF₁₀(Pri-MA) = 102.0 BF₀₁(Rep-MA) = 4.29	BF₀₁(Pri-Rep) = 4.91 BF₀₁(Pri-MA) = 9.11 BF₀₁(Rep-MA) = 9.93
paired samples *t*-test (one-sided)				
sample size	BF₁₀ = 9.19 × 10¹¹	BF₁₀ = 1.16 × 10⁶	BF₁₀ = 5.63 × 10¹³	BF₁₀ = 2254
main IV	BF₀₁ = 1.33	BF₀₁ = 8.52	–	–
guilt ratings				
ANOVA				
sample size	BF₀₁ = 1.91	BF₀₁ = 9.34	BF₀₁ = 12.36	BF₀₁ = 12.44
main IV	BF₀₁ = 8.41	BF₀₁ = 8.94	BF₀₁(Pri-Rep) = 7.78 BF₀₁(Pri-MA) = 8.80 BF₀₁(Rep-MA) = 11.80	BF₁₀(Pri-Rep) = 2.31 BF₀₁(Pri-MA) = 11.16 BF₁₀(Rep-MA) = 1.18
paired samples *t*-test (one-sided)				
sample size	BF₀₁ = 1.16	BF₀₁ = 4.68	BF₀₁ = 4.92	BF₀₁ = 13.11
main IV	BF₀₁ = 3.23	BF₀₁ = 3.95	–	–

#### 3.1.1. Study 1: preregistration × sample size

*Doctorate population:* Analyses were conducted using Bayesian repeated-measures ANOVAs with preregistration status (preregistered versus no information) and sample size (low versus high) as within-subject factors. For confidence ratings, the best-fitting model included the main effect of sample size (BF_10_ = 4.44 × 10¹¹ compared to the null model), providing conclusive evidence. Bayes factors likewise indicated support for an effect of sample size (BF_10_ = 3.76 × 10^20^). This served as our manipulation check and showed that participants reported higher confidence in the research finding when the presented study included a large sample (*M* = 69.85, 95% CI [66.32, 73.39]) compared to those including small samples (*M* = 43.09, 95% CI [37.78, 48.41]; *η*² = 0.48). This effect also held in an additional one-sided Bayesian paired samples *t*-test (BF_10_ = 9.19 × 10^11^). The preregistration factor was not supported (BF_01_ = 2.65, BF_01_ = 1.33 in a one-sided paired samples *t*-test; preregistered: *M* = 57.91, 95% CI [54.23, 61.58]; no information: *M* = 55.04, 95% CI [50.95, 59.13]). Thus, although not conclusively supporting the null, we did not find evidence that participants reported more confidence in the research finding when the study was preregistered (versus no information).

For guilt judgements, the best model explaining the data was the null model, indicating that neither sample size nor preregistration status improved model fit. Contrary to the hypotheses, the evidence did not support either direction of effect for sample size (BF_01_ = 1.91, BF_01_ = 1.16 in one-sided test; did not reach the conclusive threshold). For preregistration, there was convincing evidence for the null over the alternative hypothesis in the ANOVA (BF_01_ = 8.41) but inconclusive evidence when testing our one-sided expectation (BF_01_ = 3.23). Hence, contrary to our expectation that higher methodological quality would lead to lower guilt ratings, we did not find robust guilt judgement effects in the study.

*Lay population:* Once again, we conducted Bayesian repeated-measures ANOVAs with preregistration status (preregistered versus no information) and sample size (low versus high) as within-subject factors. For confidence ratings, the best-fitting model showed conclusive evidence for a main effect of sample size (BF_10_ = 6.20 × 10^5^ compared to the null model). In addition, the Bayes factor indicated strong support for an effect of sample size (BF_10_ = 3.10 × 10^9^, BF_10_ = 1.16 × 10^6^ in a one-sided paired sample *t*-test). Thus, participants reported higher confidence in research findings when the study included a large sample (*M* = 68.39, 95% CI [64.96, 71.82]) than when it included a small sample (*M* = 52.51, 95% CI [47.75, 57.26]; *η*² = 0.30). As in the doctorate population, the preregistration factor was not supported in the model (BF_01_ = 11.90; BF_01_ = 8.52 when testing a one-sided paired samples *t*-test; preregistered: *M* = 60.45, 95% CI [56.53, 64.37]; no information: *M* = 60.44, 95% CI [56.97, 63.92]), indicating that preregistration status did not affect confidence ratings. To supplement these findings, raincloud plots depicting the full distribution of confidence ratings by population and condition are provided in figure 1.

**Figure 1.**
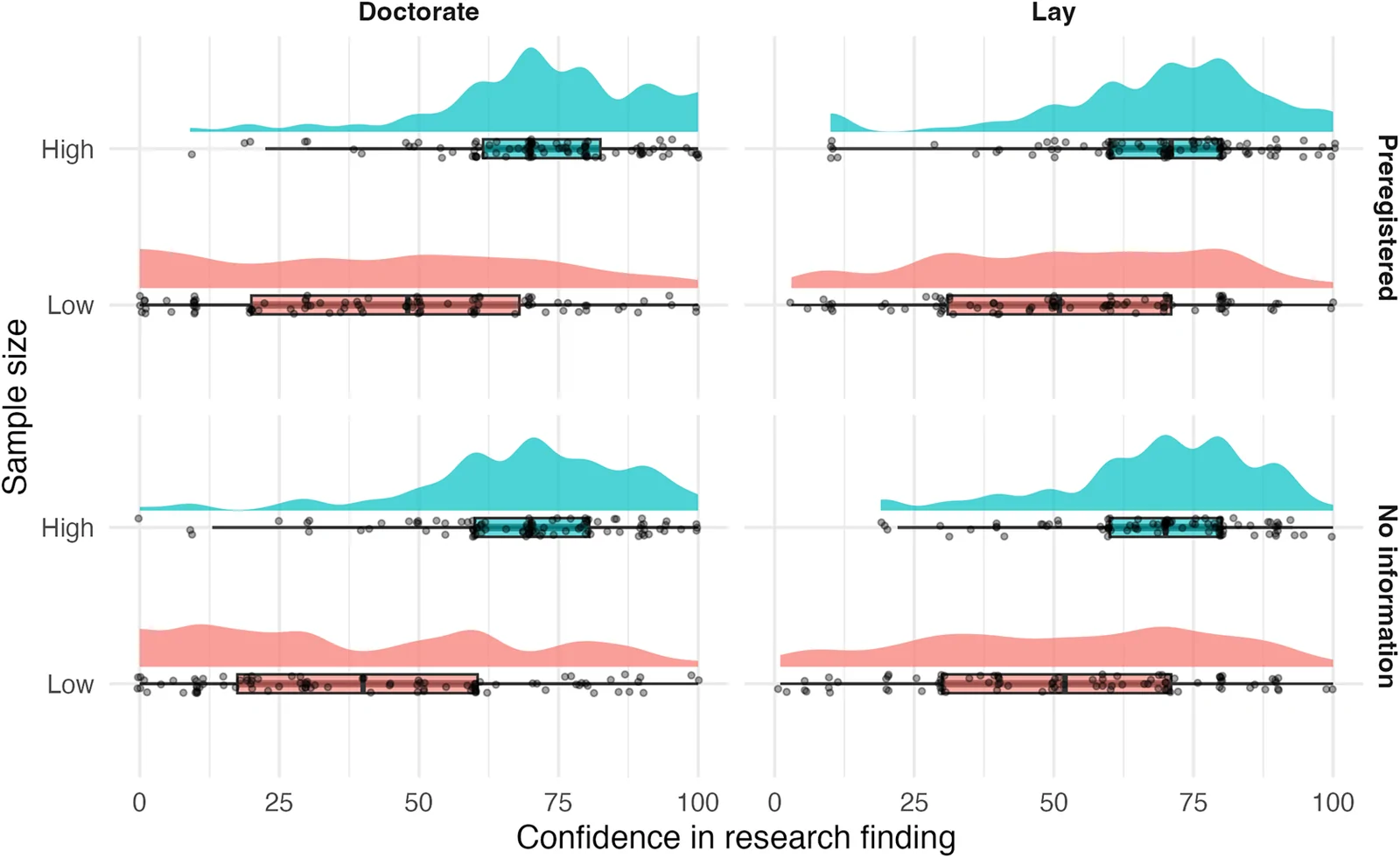
Raincloud plots of perceived research reliability by sample size, preregistration status and population.

For guilt judgements, the best-fitting model was again the null model, with no evidence that either factor improved model fit. Bayes factors supported the absence of effects for sample size (BF_01_ = 9.34; one-sided test: BF_01_ = 4.68) and preregistration (BF_01_ = 8.94; one-sided test: BF_01_ = 3.95). Hence, as in the doctorate population, participants were sensitive to sample size as an indicator of study quality in their confidence ratings but not in their legal judgements.

#### 3.1.2. Study 2: design × sample size

*Doctorate population:* Analyses were conducted using Bayesian repeated-measures ANOVAs with study design (primary versus replication versus meta-analysis) and sample size (low versus high) as within-subject factors. The best-fitting model included both main effects of study design and sample size (BF_10_ = 1.07 × 10^15^ compared to the null model), providing conclusive evidence. The Bayes factor likewise indicated conclusive evidence for an effect of sample size (BF_10_ = 5.33 × 10^30^). As expected, participants reported higher confidence in studies with large samples (*M* = 72.54, 95% CI [69.29, 75.78]) than in those with small samples (*M* = 49.83, 95% CI [45.03, 54.62]; *η*² = 0.54). The one-sided paired samples *t*-test showed similar results (BF_10_ = 5.63 × 10^13^). Confidence also increased across the evidential hierarchy, from primary studies (*M* = 57.24, 95% CI [52.94, 61.54]) to replications (*M* = 62.11, 95% CI [58.51, 65.71]) and meta-analyses (*M* = 64.19, 95% CI [60.14, 68.24]). Aligned with our hypotheses, post hoc Bayesian *t*-tests provided conclusive evidence that replication studies (BF_10_ = 6.82) and meta-analyses (BF_10_ = 102.00) were rated as more trustworthy than primary studies. There was no evidence for a difference in confidence ratings between replication and meta-analyses (BF_01_ = 4.29). The results are presented in figure 2.

**Figure 2.**
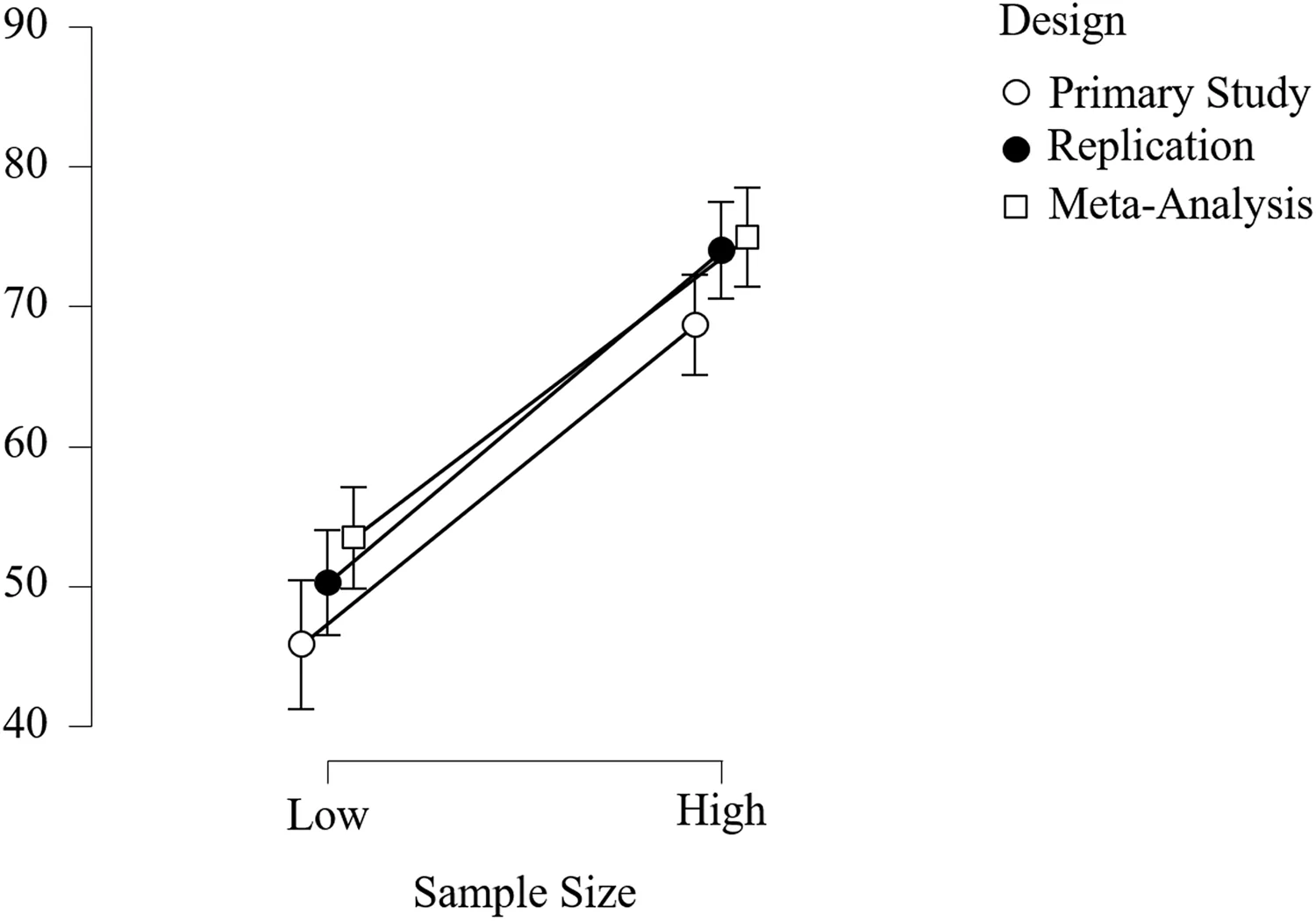
Perceived research reliability as a function of sample size and study design with 95% confidence intervals (doctorate population). Note that the *y*-axis ranges from 40 to 90 (not 0–100) to improve visibility of group differences. This scaling can visually exaggerate effect sizes.

For guilt judgements, the best-fitting model was the null model, indicating that neither study design nor sample size improved model fit. Bayes factors showed conclusive evidence for the absence of effects for sample size (BF_01_ = 12.36, BF_01_ = 4.92 for the one-sided paired samples *t*-test). For study design, all post hoc comparisons showed conclusive evidence for the null hypothesis (primary-replication: BF_01_ = 7.78, primary-meta-analysis: BF_01_ = 8.80, replication-meta-analysis: BF_01_ = 11.80). This suggests that while methodological quality cues shaped participants’ confidence in the scientific evidence, they did not influence their guilt judgements.

*Lay population:* Bayesian repeated-measures ANOVAs included study design (primary versus replication versus meta-analysis) and sample size (low versus high) as within-subject factors. The best-fitting model included sample size only (BF_10_ = 1.22 × 10^3^ compared to the null model), and the sample size Bayes factor confirmed this pattern (BF_10_ = 504 800, BF_10_ = 2254 for one-sided paired samples *t*-test). Thus, participants rated studies with large samples (*M* = 64.52, 95% CI [60.59, 68.46]) as more reliable than those with small samples (*M* = 54.24, 95% CI [49.53, 58.95]; *η*² = 0.19). However, in contrast to the hypothesis and the doctorate population, we found conclusive evidence for an absence of effect for study design (i.e. primary-replication: BF_01_ = 4.91, primary-meta-analysis: BF_01_ = 9.11, replication-meta-analysis: BF_01_ = 9.93), suggesting that lay participants did not find the research more reliable when presented with studies that were replications or meta-analyses, compared to primary studies. Similarly to the above, raincloud plots depicting the full distribution of confidence ratings by population and condition are provided in figure 3.

**Figure 3.**
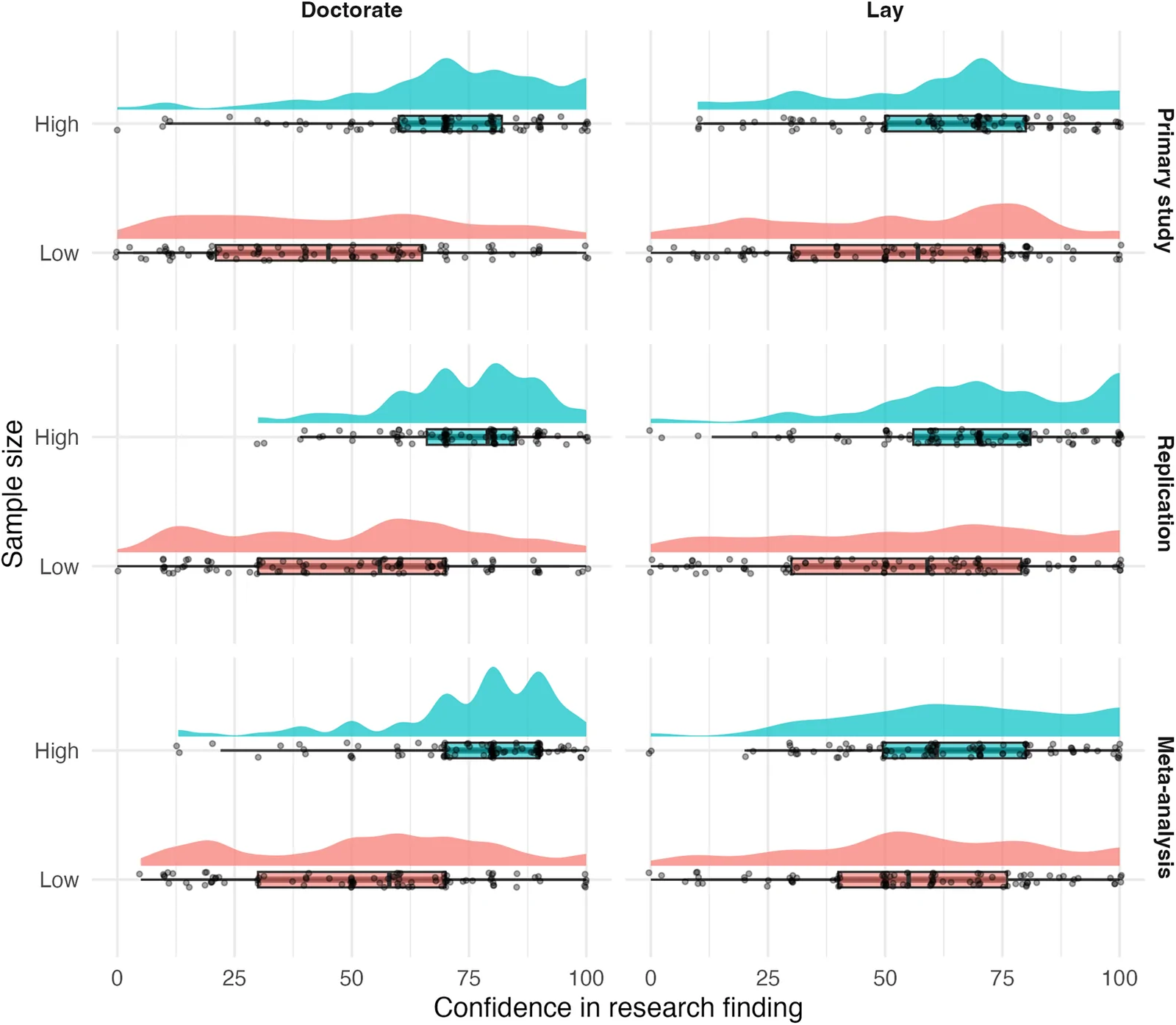
Raincloud plots of perceived research reliability by sample size, study design and population.

Finally, for guilt judgements, the best-fitting model was again the null model, indicating that neither study design nor sample size affected participants’ guilt decisions. We found conclusive evidence for the absence of a sample size effect (BF_01_ = 12.44; one-sided paired samples *t*-test: BF_01_ = 13.11). The evidence for study design differences in guilt judgements was more inconclusive (i.e. primary-replication: BF_10_ = 2.31, primary-meta-analysis: BF_01_ = 11.16, replication-meta-analysis: BF_10_ = 1.18). Still, overall, this indicated that lay participants were sensitive to sample size as a cue of evidential strength in their reliability assessments, but this did not translate into differential guilt decisions.

### 3.2. Exploratory analyses

To contextualize the null effects on guilt judgements, and to examine whether the exonerating research evidence had any general influence on guilt decisions independent of methodological quality, we examined overall guilt means collapsed across conditions. Across all four studies, average guilt ratings approximated the midpoint of the scale (Study 1 doctorate: *M* = 46.05, *s.d.* = 14.43; Study 1 lay: *M* = 49.74, *s.d.* = 12.60; Study 2 doctorate: *M* = 44.75, *s.d.* = 12.55; Study 2 lay: *M* = 50.45, *s.d.* = 13.46), suggesting that participants were broadly uncertain about the suspect's guilt rather than showing a clear directional shift towards innocence or guilt overall. We additionally examined whether participants who found the research more reliable also tended to judge the suspect as less guilty, by correlating participant-level mean confidence and mean guilt ratings within each study and population. Across all four studies, Bayesian correlations provided evidence, although not always convincingly, for the null over the alternative hypothesis (Study 1 doctorate: BF_01_ = 4.02, *r* = 0.03; Study 1 lay: BF_01_ = 1.80, *r* = −0.14; Study 2 doctorate: BF_01_ = 3.88, *r* = 0.04; Study 2 lay: BF_01_ = 2.99, *r* = −0.09), further corroborating the dissociation between research reliability judgements and guilt judgements observed in the confirmatory analyses.

## 4. Discussion

In response to the replication crisis, psychology has undergone a profound methodological transformation. Best and Open Science practices were introduced to strengthen research transparency, reproducibility and rigour and ultimately trust in science. Yet, despite their growing prominence, it remains unclear how these norms are perceived and applied by different audiences—and whether they translate into judgements of reliability or decision-making in legal contexts. Building on earlier research (e.g. [21,27,28]), the present study addressed this question by examining how individuals with differing levels of research experience (i.e. doctoral graduates and laypersons) evaluate the reliability of psychological findings as a function of methodological quality indicators and whether these evaluations extend to downstream guilt decisions. Using a vignette paradigm situated in the domain of false memory research, participants assessed fictitious cases and rendered verdicts of guilt. False memory research provided an ideal context for this inquiry: As psychological evidence in this field can carry real-world consequences in court, it offers a meaningful test case for how contemporary research norms shape perceptions of scientific reliability and applied reasoning.

There are four main findings. First, sample size consistently emerged as an influential predictor of perceived research reliability. Second, doctorate but not lay participants showed sensitivity to evidential strength—rating replications and meta-analyses as more reliable than primary studies. Third, preregistration did not influence perceived reliability in either group. Lastly, *none* of the study factors (i.e. sample size, design and preregistration) influenced guilt judgements in either group. This highlights that while people can recognize certain indicators of quality when judging scientific reliability, this sensitivity does not translate into decisions about guilt. We discuss these results in more detail below.

First, in line with prior work (e.g. [21]), participants consistently rated studies with large samples as more trustworthy than those with small samples. This large effect emerged robustly across both research-familiar doctorate and lay populations, suggesting that sample size functions as a broadly understood heuristic for evidential strength and aligns with long-standing conventions in scientific reasoning (e.g. [39]). However, the present manipulation contrasted markedly different sample sizes (approx. 10 versus 1000 participants). This deliberate choice served as a manipulation check to ensure sensitivity to classical methodological quality cues, but it may have also amplified perceived differences and inflated effect sizes. Moreover, very large samples can produce compelling statistical evidence for effects of limited practical relevance—a phenomenon often discussed as the large-sample paradox (e.g. see [40]). Future studies could therefore examine more nuanced variations to assess whether this heuristic persists under subtler and more realistic conditions. Nevertheless, the current findings show that sample size might act as an interpretable and influential cue of reliability, even outside of specialist audiences.

Second, doctorate-level participants rated replication studies and meta-analyses as more reliable than primary studies. This likely reflects the familiarity of doctorate-level participants with the evidential hierarchy and an understanding of the scientific process as cumulative and self-corrective [41]. Replications and meta-analyses are designed to test robustness and generalizability [42,43] and thus may intuitively signal higher evidential strength to research-trained individuals. Notably, neither replication nor meta-analysis inherently guarantees methodological superiority. Replications can still yield false positives or false negatives (e.g. [44]), and their quality depends on adherence to rigorous protocols, preregistration and power (e.g. [45]). Similarly, the validity of a meta-analysis depends on the quality of its studies, analytic transparency and bias correction procedures [46,47].

Nonetheless, since the replication crisis prompted widespread reform—including greater transparency, preregistration and resource sharing—contemporary replication and meta-analytic efforts may be expected to, on average, embody higher methodological standards than earlier work. This may explain why doctorates, who are likely to be attuned to these developments, have judged such designs as more reliable. In addition, both replication and meta-analysis represent systematic re-evaluations of existing knowledge, inherently providing an additional layer of confirmation beyond single studies. In the present studies, where replication and meta-analytic findings consistently aligned with the primary results, this convergence may have been perceived as providing stronger and more cumulative support for the same conclusion—an impression that could further enhance reliability judgements among research-familiar audiences [41,48], even if the objective epistemic value of such convergence depends on the methodological quality of the underlying studies.

The absence of a corresponding effect among lay participants suggests that understanding the evidential hierarchy may require domain-specific knowledge. Whereas sample size can be grasped more intuitively, the epistemic implications of replication and meta-analysis are less self-evident without prior familiarity with research methodology, aligning with previous research (e.g. [28,49]). This divergence underscores the gap between professional and public interpretations of methodological rigour and suggests that methodological reforms may only enhance perceived reliability among audiences who already know and understand their significance.

Third, contrary to our expectations, preregistration did not influence reliability ratings in either population. Two complementary explanations may account for this finding. One is that preregistration itself does not guarantee study quality but rather allows others to evaluate how severely a claim has been tested [13]. Vague or incomplete preregistrations may still fail to constrain researcher degrees of freedom [50,51], and even well-executed preregistrations do not prevent methodological biases elsewhere in the process. Indeed, recent evidence suggests that preregistered studies in psychology may not be more replicable than non-preregistered ones [14]. If preregistration does not demonstrably improve study quality in practice, it may be reasonable not to treat it as a meaningful signal of research reliability. Nevertheless, we argue that on average, preregistration fosters planning transparency, discourages selective reporting and reduces file drawer studies [12]. Further, variability in practice quality alone cannot explain our null effect, since study design, likewise variable, affected reliability among doctorates.

Another plausible explanation is that preregistration remains relatively uncommon compared to other practices within psychological subfields (e.g. [3]). If the practice itself is unfamiliar or uncommon even to researchers, its broader informational value as a quality signal may be weak. This interpretation aligns with broader findings that practices enhance reliability primarily when their meaning is explicitly communicated (e.g. via open-science badges [28]). Song *et al.* [29] reported a comparable effect for Open Science more generally, but their manipulation bundled preregistration with open data and materials sharing rather than isolating it. Moreover, in two of their three studies, conceptual definitions were delivered immediately before the exposure to the research information. Our glossary, by contrast, explained key concepts alongside distractor terms in the beginning of the session decoupled from any specific vignette. Taken together, these results suggest that preregistration may not yet function as an isolated reliability cue, reflecting a gap between its methodological importance and perceived relevance.

Lastly, despite effects on perceived research reliability, none of the methodological quality cues influenced guilt judgements in the current studies. The descriptive pattern of guilt ratings across both populations and studies approximated the midpoint of the scale, suggesting that participants did not hold strong opinions about the suspect's guilt overall, and confidence in the research findings was not associated with guilt ratings across any of the four studies. Because presentation order was randomized across participants, any influence of repeated exposure to exonerating evidence on task engagement would be expected to introduce noise rather than systematically bias guilt judgements in any particular condition. This makes it unlikely that the absence of a reliability-to-verdict pathway reflects hypothesis awareness or non-compliance with the task. More substantively, guilt evaluations appeared to rely more on the specifics of the case or intuitions than on methodological considerations, which is plausible given that scientific findings describe general probabilities rather than case-specific certainties. Participants may have perceived the evidence as insufficiently diagnostic to determine an individual's guilt or innocence.

This pattern is not unique to the present study. Forensic psychology literature has documented that legal decision makers’ sensitivity to the quality of expert evidence does not reliably translate into their downstream legal judgements: judges’ admissibility decisions and jurors’ evaluations of expert testimony have been found to be insensitive to manipulated variations in scientific quality [22,23]. Chorn & Kovera [52], for instance, found that although attorneys’ ratings of an expert's scientific quality were sensitive to variations in test reliability, this sensitivity did not extend to their decisions about whether to challenge the evidence, and judges’ admissibility decisions were unaffected altogether. More recently, LaBat *et al.* [53] found that even after an intervention successfully improved jurors’ sensitivity to the quality of forensic testimony, their ratings of the physical evidence and their verdicts remained unchanged.

More broadly, this dissociation between reliability and guilt judgements aligns with related research on science communication. Evidence from public health and risk communication suggests that scientific information is more likely to shape judgements and behaviour when it is conveyed by trusted sources, framed as consistent with prevailing scientific or social norms and aligned with recipients’ prior beliefs or intuitions (e.g. see [20,54]). From this perspective, methodological rigour alone may be insufficient to influence decision-making unless accompanied by communication strategies that facilitate the translation of scientific evidence into case-specific reasoning.

In the current study, several limitations warrant consideration. The online vignette format, though controlled and efficient, limited ecological validity, particularly for the guilt judgements. The short, neutral and decontextualized case summaries and systematic structure (evidence always favouring innocence) likely constrained participants’ ability to engage in naturalistic jury-like reasoning. This may have encouraged cautious or ambivalent responses, treating the research findings as informational rather than decisive evidence. Such a response tendency may have been particularly consequential for the null effects observed on guilt judgements. We therefore emphasize that the null effect on guilt judgements should not be read as evidence that methodological quality is irrelevant to legal decisions. Several features of the design could plausibly produce this pattern independently of participants’ reasoning: the absence of consequences, deliberation and adversarial argumentation; the uniform direction of the evidence, which removed any conflict for participants to resolve; the absence of competing case evidence against which the research could be weighed; and a single-item guilt measure that may have been insensitive to small shifts. Whether methodological quality cues influence verdicts when these elements are present remains an open empirical question that the present design cannot answer. Additionally, the within-subject design means participants evaluated methodological features across multiple vignettes in direct comparison with one another, which may not reflect how jurors encounter scientific evidence in court. This comparative context may have shaped reliability judgements in ways that differ from the more absolute evaluations a juror would make. Future research could address this by using immersive or interactive paradigms, such as mock trials, virtual reality simulations or deliberative group designs, to examine how methodological information shapes real-time legal reasoning.

Moreover, while we distinguished between doctoral and lay populations, the doctoral sample was recruited via Prolific's education filter identifying completed degrees without specifying the field of study. Doctoral training across disciplines plausibly confers general familiarity with research methodology, but the degree of such familiarity likely varies considerably: doctorates in empirical disciplines may be more attuned to methodological quality cues than those trained in fields with less quantitative emphasis. More broadly, actual expertise may have varied, and their familiarity with research methods or legal contexts was not fully characterized. Future work should therefore compare specialized subgroups (e.g. practising researchers in empirical fields, legal professionals or jury-eligible citizens) to examine how domain expertise may moderate sensitivity to methodological quality.

Finally, the present study focused on the three indicators: *sample size*, *study design* and *preregistration*. This represents only a subset of Open Science and best-practice dimensions. Future studies could expand this framework to include material, data, code sharing or other contemporary practices (e.g. open peer review, collaborative expert efforts) to provide a more comprehensive understanding of how the reforms in the field influence perceived reliability and subsequent decision-making.

Taken together, in an era increasingly shaped by misinformation, scientific scepticism and polarized discourse [55,56], the ability to recognize reliable evidence is essential for informed judgement and responsible decision-making—particularly in domains with real-world consequences. The present findings speak directly to this challenge: even under controlled and simplified conditions, methodological features, such as sample size and study design, influenced how people evaluated psychological evidence. This may suggest that sensitivity to research quality is not limited to trained scientists, and that targeted education about research norms could meaningfully enhance the quality of evidence evaluation. Our findings therefore underscore the importance of bridging the gap between scientific practice and public understanding, so that both scientific and public audiences are better equipped to interpret and use scientific evidence responsibly in legal decision-making.

### 4.1. Constraints on generality

The present findings should be interpreted with several constraints on generality in mind. First, although we deliberately sampled two populations with differing levels of research literacy (doctoral graduates and laypersons), these groups do not exhaust the range of legal decision makers and may thus not generalize to other groups such as legally trained professionals (e.g. judges and attorneys), for whom institutional norms, group dynamics and accountability pressures may alter how scientific evidence is interpreted and applied.

Second, the materials relied on brief, abstract-like research summaries embedded in short written vignettes. This format allowed precise experimental control over methodological features but necessarily simplified how scientific evidence is encountered in real legal settings. As noted by a reviewer, in common law jurisdictions, academic research articles cannot be adduced directly as evidence; they would typically be introduced through expert witness testimony. Beyond this procedural simplification, expert testimony is also often conveyed orally, contextualized by cross-examination and embedded within competing narratives. While we expect sensitivity to reliability cues to generalize across formats, the null effects on guilt judgements may be specific to this highly constrained presentation. Therefore, future work should engineer scenarios and settings, which more accurately reflect the procedural realities of how psychological science enters legal proceedings.

Third, the procedural structure of the task may have limited generalizability. Participants evaluated multiple cases in a within-subject design and made independent judgements without deliberation, feedback or consequences. Legal decisions in the real world are typically one-shot, socially embedded and outcome-relevant. Thus, the observed dissociation between reliability judgements and legal decisions may differ in settings involving collective deliberation, adversarial argumentation or explicit decision rules.

Finally, the context of false memory research, while legally relevant, represents only one domain in which psychological science informs legal outcomes. The degree to which these findings generalize to other domains (e.g. risk assessment, eyewitness identification procedures and forensic evidence) remains an open question.

## Data Availability

All data, analysis code and research materials are available on the Open Science Framework [36]. Studies were preregistered (including the analysis plan) prior to data collection at https://osf.io/68gte/overview (doctorate population) and https://osf.io/ntkw5/overview (lay population).

## Footnote

^1^
The G*Power calculation assumed a correlation among repeated measures of *r* = 0.50, which reflects a moderate-to-high assumption common in within-subjects designs. Post hoc inspection of the observed data revealed mean inter-item correlations ranging from 0.20 to 0.41. Specifically, under the observed correlations for Study 1 (*r* ≈ 0.20–0.30), the required sample would have been 112–128 participants approximately. Our actual samples of 89–91 participants therefore fell below these recalculated thresholds. Post hoc sensitivity analyses using the final sample sizes and observed inter-item correlations indicate that the studies could detect effects of *f* = 0.15–0.17 at 80–90% power for reliability ratings and *f* = 0.15–0.18 at 77–87% power for guilt ratings, using the omnibus repeated measures ANOVA. For individual pairwise comparisons, the minimum detectable effect was *d* ≈ 0.30 at 80% power across all four studies. While these sensitivities are somewhat below the originally intended 90% threshold, two factors limit the practical impact of underpowering: (i) our primary analyses were Bayesian rather than frequentist and (ii) the effect size of *f* = 0.15 was itself conservative relative to the Bottesini *et al.* [21] benchmark.
